# Enhanced light absorption of amorphous silicon thin film by substrate control and ion irradiation

**DOI:** 10.1186/1556-276X-9-173

**Published:** 2014-04-09

**Authors:** Fangda Yuan, Zhengcao Li, Tianci Zhang, Wei Miao, Zhengjun Zhang

**Affiliations:** 1 Key Laboratory of Advanced Materials, School of Material Science and Engineering, Tsinghua University, Beijing 100084, China

**Keywords:** Enhanced light absorption, Silicon, Irridiation

## Abstract

Large-area periodically aligned silicon nanopillar (PASiNP) arrays were fabricated by magnetic sputtering with glancing angle deposition (GLAD) on substrates coated by a monolayer of close-packed polystyrene (PS) nanospheres. The structure of PASiNP arrays could be manipulated by changing the diameter of PS nanospheres. Enhanced light absorptance within a wavelength range from 300 to 1,000 nm was observed as the diameter of nanopillars and porosity of PASiNP arrays increased. Meanwhile, Xe ion irradiation with dose from 1 × 10^14^ to 50 × 10^14^ ions/cm^2^ was employed to modify the surface morphology and top structure of thin films, and the effect of the irradiation on the optical bandgap was discussed.

**PACS code:**

81.15.Cd; 78.66.Jg; 61.80.Jh

## Background

With the development of society, problems of energy crisis and environment pollution have been of further concern. In order to maintain sustainable growth, a clean and renewable energy source is urgently required. Among all new types of energy sources, solar energy is the most promising one for it is safe, cheap, inexhaustible, and environment-friendly.

In 1976, Carlson and Wronski [1] invented a new type of thin film solar cell that utilized amorphous silicon (a-Si) deposited from a glow discharge in silane (SiH_4_) and achieved a power conversion efficiency of 2.4% in AM-1 sunlight. After that, silicon thin film solar cells have been widely investigated in different ways and methods [2]. Compared with conventional solar cell, amorphous silicon thin film solar cell is low cost and could be deposited on various substrates such as glass, stainless steel, ceramic plate, and plastic [3].

Studies focused on textured surface showed that it can improve absorption by reducing reflection. Textured surface can be conventionally obtained by either dry or wet ion etching [4-7]. In 2011, Wong and Yu [8] simulated a nanopillar-array-textured surface and came to a conclusion that it may enhance light absorption and increase the efficiency of the silicon-based solar cell.

The effects of low-energy heavy ion irradiation on silicon thin film have been systematically studied during the past 50 years. During the irradiation, some traditional defects were generated; however, latent tracks, amorphous transition, or other special effects were not observed [9,10]. Enhanced light absorption was obtained in works on n-type crystal silicon irradiated by high-energy Xe ion [11], which provided a promising method for the modification of amorphous silicon thin film.

In this research, we coated a polystyrene (PS) sphere monolayer on glass substrate and fabricated silicon thin film via magnetic sputtering with glancing angle deposition (GLAD) in order to achieve periodically aligned silicon nanopillar (PASiNP) arrays. The influences of silicon nanopillar diameter and Xe ion irradiation on the light absorption of thin film were studied. The mechanism of ion irradiation was also discussed.

We replicate this nanostructure by magnetic sputtering deposition with its advantage of controllable fabrication, and an expected enhancement in light absorption was observed.

## Methods

Glasses were first cut into squares of about 3 × 3 cm^2^ in size and then thoroughly cleaned with acetone in an ultrasonic bath for 20 min. After washing off the residual acetone by deionized water, they were cleaned with ethanol in an ultrasonic bath for another 20 min. The glasses were immersed in H_2_SO_4_-H_2_O_2_ solution (3:1, *v*/*v*) for 8 h and then cleaned with deionized water in an ultrasonic bath for 30 min and with NH_3_-H_2_O_2_-H_2_O solution (1:1:3, *v*/*v*) for another 30 min. After that, glasses with hydrophilic surfaces were obtained [12].

PS nanospheres with different diameters of 200, 500, and 1,000 nm were selected here. Ten microliters of PS solution (10 wt. %) was distributed onto a piece of glass which was placed on a spin coater. The solution was spread and dried to form a monolayer. Details are shown in Table 1.

**Table 1:** Details of spinning and drying

**Diameter of PS nanosphere (nm)**	**Rate and time of spinning (r/min × min)**	**Rate and time of drying (r/min × min)**
200	120 × 1	350 × 4
500	120 × 1	250 × 4
1000	120 × 1	150 × 4

The silicon thin film was then deposited on the substrates via magnetic sputtering in argon atmosphere at 1.5 Pa for 90 min at a deposition angle of about 80°. The sputtering power was 90 W and the voltage was 0.5 kV.

Afterwards, films deposited on the 200-nm PS nanosphere monolayer were irradiated by 200-keV Xe ion with doses of 1 × 10^14^, 5 × 10^14^, 10 × 10^14^, and 50 × 10^14^ ion/cm^2^, in order to investigate its influence on the light absorption of thin film.

The morphology of films was observed by scanning electron microscopy (SEM). The X-ray diffraction (XRD) patterns were tested by Rigaku X-ray analytical instrument (Rigaku Corporation, Tokyo, Japan). The transmittance (*T*) and reflectance (*R*) spectra within the wavelength range from 300 to 1,000 nm were recorded by a UV-Vis-NIR spectrometer.

## Results and discussion

**Figure 1:**
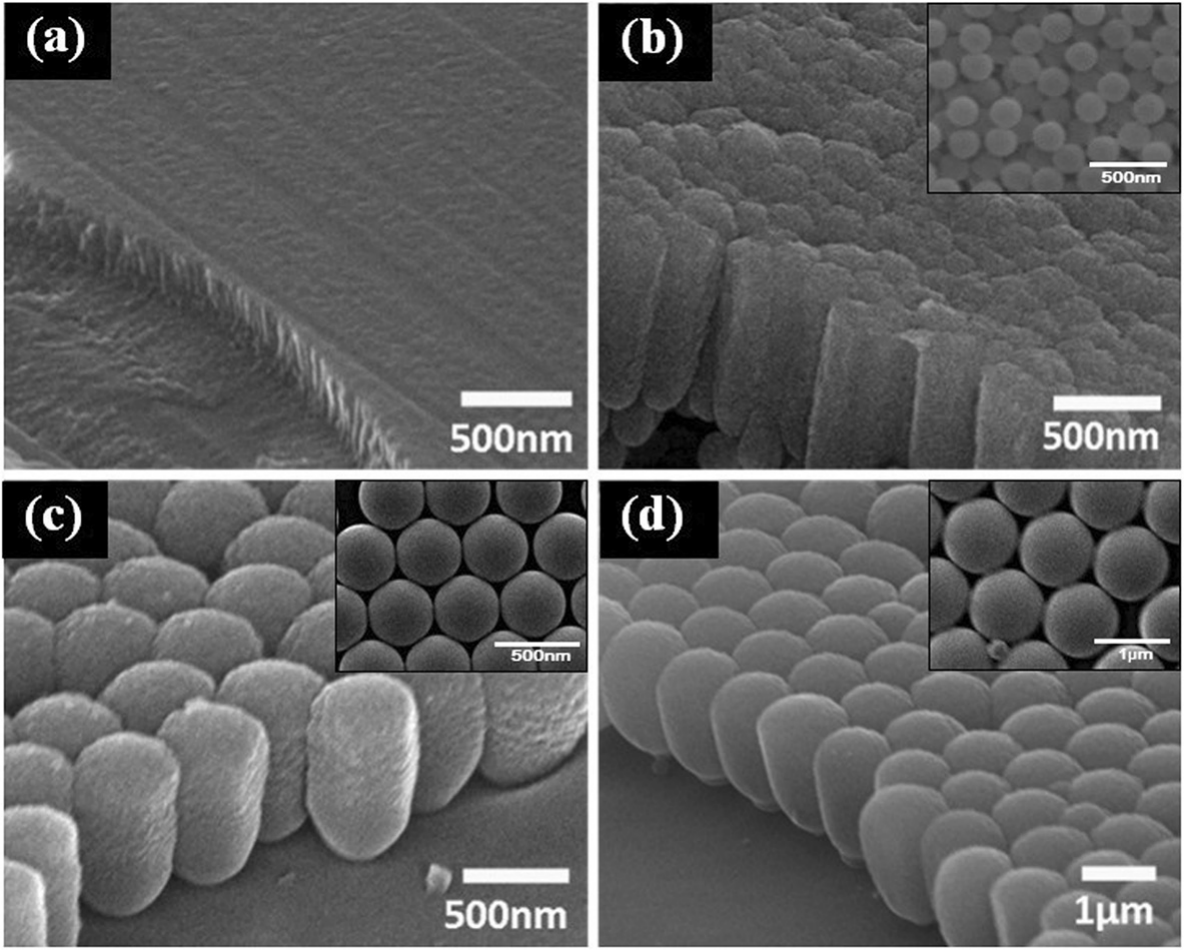
**Cross-sectional view of silicon nanopillar arrays deposited on substrates coated by PS nanospheres with different diameters. (a)** 0 (plain glass), **(b)** 200 nm, **(c)** 500 nm, and **(d)** 1,000 nm. The insets show the morphology of the corresponding PS nanosphere monolayer.

The morphology of the PS nanosphere monolayer was shown in the insets of Figure 1. PS nanospheres were self-assembled into a monolayer, and a highly ordered area of about 50 μm^2^ was obtained. For the 500- and 1,000-nm PS nanosphere monolayers, the arrays were nearly hexagonal and close-packed. However, for the 200-nm PS nanosphere monolayer, the distribution was less regular and there were many vacancies and dislocations due to the kinetic limitations during the drying process [13,14].

After 90 min of deposition, films with thickness of about 700 nm were obtained, as shown in Figure 1. They were marked after their deposition time and the diameter of PS nanospheres as 90-0, 90-200, 90-500, and 90-1000; 0 represented the plain glass, which was used for comparison. For the films deposited on patterned substrates, owing to GLAD and shadowing effect, each nanosphere leads to the formation of one nanopillar. The size of nanopillars is determined by the diameter of the PS nanospheres beneath, and the nanopillar arrays replicate the close-packed pattern of the monolayer. Nanopillars separate from each other, and porosity rises as the diameter increases.

**Figure 2:**
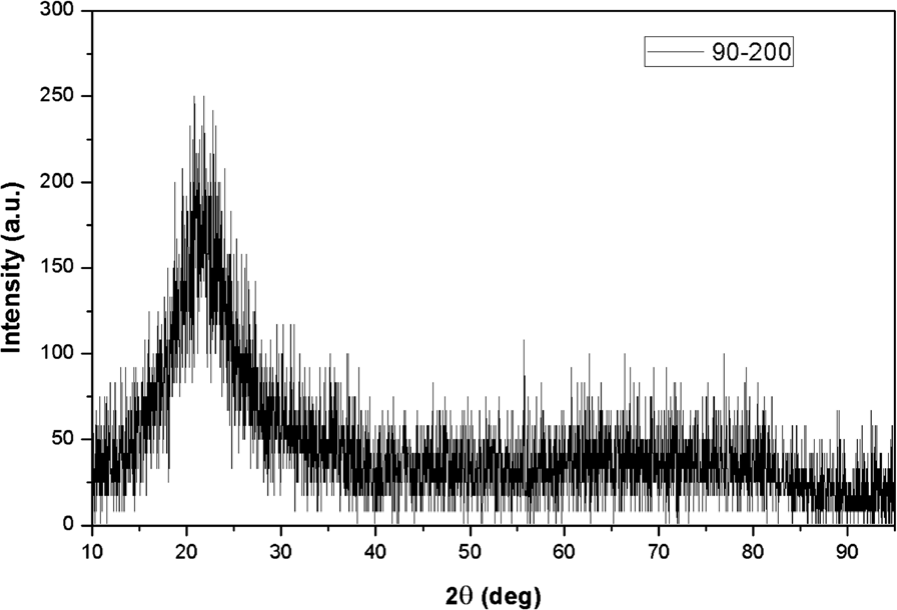
XRD patterns of films deposited on substrates coated by PS nanospheres with diameter of 200 nm.

Silicon atoms were randomly deposited on the PS nanosphere monolayer during the GLAD process, and thin films were not annealed afterwards and thus cannot develop into crystals. The XRD pattern of sample 90-200 is shown in Figure 2. No typical peak for crystal silicon can be found in this pattern, as well as for the other three, which indicates that these films are composed of amorphous silicon.

**Figure 3:**
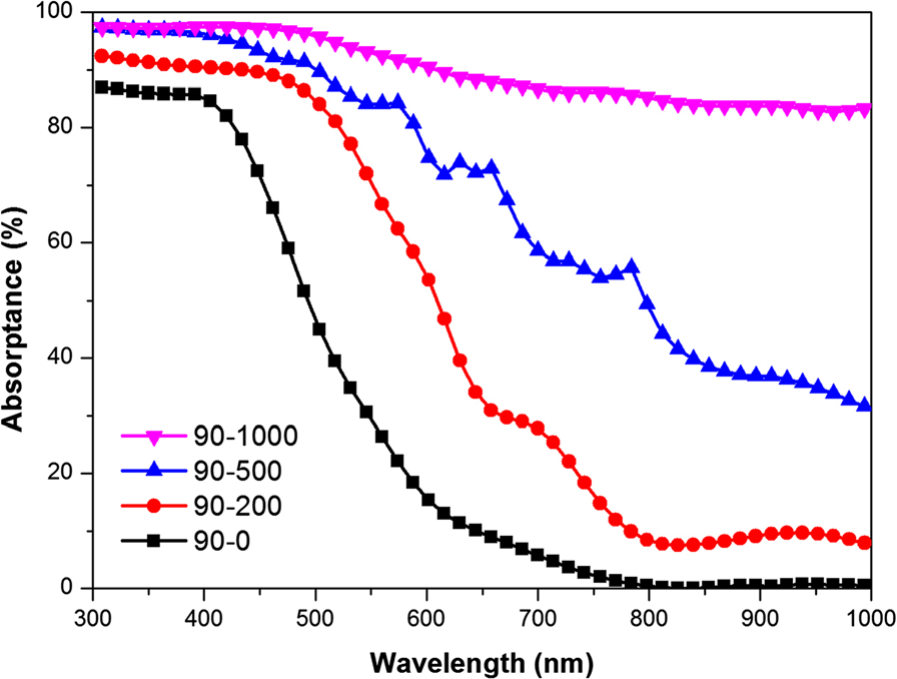
Absorptance spectra of films deposited on substrates coated by PS nanospheres with different diameters.

The absorptance (*A*) spectra shown in Figure 3 was calculated by Equation 1.

(1) A=1-T-R

The film deposited on plain glass showed poor absorptance of lower than 10%, especially within a wavelength above 800 nm. In comparison, the absorptance of films deposited on patterned substrates enhances appreciably to more than 80%. As the diameter of the nanopillar increases, the absorptance of the corresponding film rises within the whole wavelength range. The positive correlation between absorptance and diameter can be attributed to the increasing porosity of the nanostructure, which extensively lengthens the path of incident light and enhances the absorptance [8].

In order to evaluate the optical bandgap of the thin film, the Tauc formula was utilized [15].

(2) $${\left(\mathrm{\alpha h\nu}\right)}^{n}=A\left(\mathrm{h\nu}-E_{g}\right)$$

(3) $$\alpha =\ln\left(T\right)/d$$

**Figure 4:**
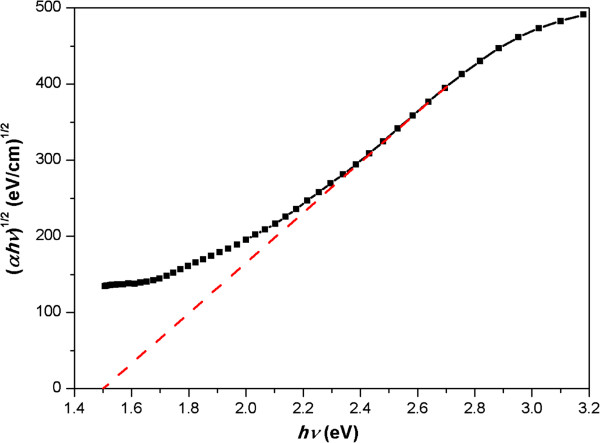
**Schematic diagram of Tauc plot.** Tauc plot was used to measure the optical bandgap of the film deposited for 90 min on a substrate coated by 1,000-nm PS nanospheres.

In Equation 2, *α* is the calculated absorption coefficient of the film which can be derived from Equation 3, *d* is the thickness of film and it was set as 700 nm here, *hv* is the energy of photon, *A* is a constant, *n* is 1/2 for indirect band material in this case, and *E*_*g*_ is the optical bandgap. We extrapolate the linear part of the (*αhν*)^1/2^ - *hν* plot to the *X*-axis, and the intercept is regarded as the calculated optical bandgap. The schematic diagram and results are shown in Figure 4 and Table 2, respectively.

**Table 2:** The optical bandgap of thin films as deposited

	**Diameter (nm)**			
	**0**	**200**	**500**	**1,000**
***E***_***g ***_**(eV)**	2.10	1.83	1.77	1.50

The reduction of optical bandgap is in accordance with the increase of absorptance. A material can only absorb photons with energy higher than its bandgap, so optical bandgap holds the essence of light absorption and the absorptance depends straightly on optical bandgap. The manipulation of optical bandgap would have direct influence on absorptance.

**Figure 5:**
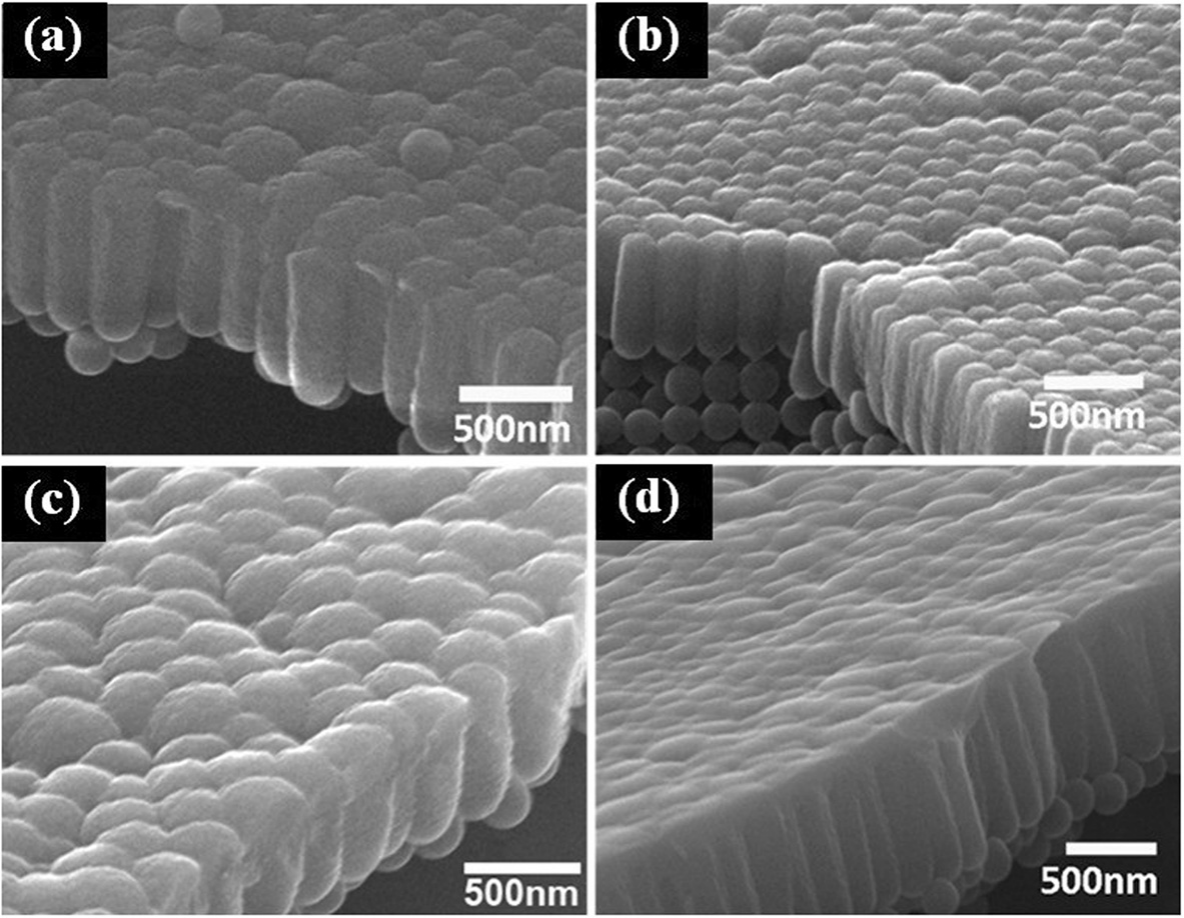
**The cross-sectional views of irradiated films with different doses. (a)** 1 × 10^14^ ions/cm^2^, **(b)** 5 × 10^14^ ions/cm^2^, **(c)** 10 × 10^14^ ions/cm^2^, and **(d)** 50 × 10^14^ ions/cm^2^.

To investigate the influence of ion irradiation on the optical bandgap of amorphous silicon thin film, films deposited on the 200-nm PS nanosphere layer were irradiated by 200-keV Xe ion with doses of 1 × 10^14^, 5 × 10^14^, 10 × 10^14^, and 50 × 10^14^ ions/cm^2^. The cross-sectional views of irradiated film are shown in Figure 5.

In the view of the original film shown in Figure 1b, silicon nanopillars are separated from each other. After ion irradiation, the top part of silicon nanopillars melted and recrystallized during the process. With the increasing of irradiation dose, the top part of the silicon nanopillars tends to connect with its neighbors, while the bottom part remains unaffected.

The optical bandgap of thin film after the irradiation was also calculated, as shown in Table 3. The optical bandgap decreases rapidly as the irradiation dose rises from 0 to 10 × 10^14^ ions/cm^2^. After that, as the irradiation dose rises from 10 × 10^14^ ions/cm^2^ to 50 × 10^14^ ions/cm^2^, it gradually levels off.

**Table 3:** Optical bandgap after irradiation

	**Irradiation dose (10**^**14 **^**ions/cm**^**2**^**)**			
	**1**	**5**	**10**	**50**
***E***_***g ***_**(eV)**	1.64	1.52	1.46	1.42

**Figure 6:**
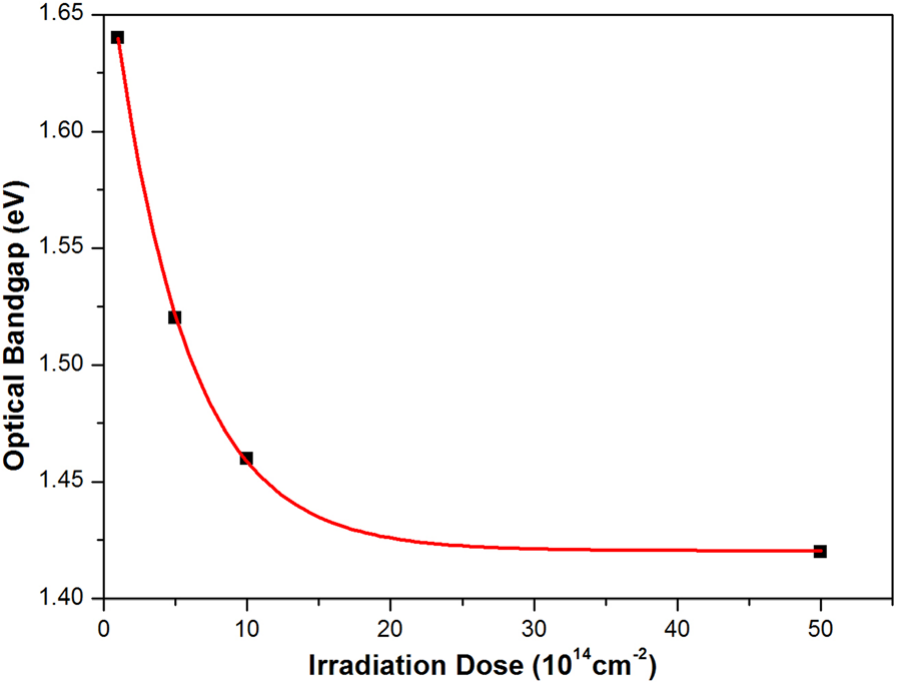
The negative exponential relation between the optical bandgap and the irradiation dose.

As shown in Figure 6, ion irradiation has distinct influence on the optical bandgap of the original film, but it may lead to a limitation as the irradiation dose increases. The optical bandgap exponential decays with the irradiation dose, and the fitting formula of the curve is $E_{g}=0.2662\times e^{-\frac{\mathrm{Dose}}{5.1426}}+1.4205$. Previous research showed that the optical bandgap decreased as the grain size of silicon expanded [16], which suggests that a possible recrystallization mechanism happened during the ion irradiation process.

## Conclusions

We prepared self-assembled monolayers of PS nanospheres and fabricated periodically aligned silicon nanopillar arrays by magnetic sputtering deposition. We improve the absorptance of thin film by changing the diameter of the silicon nanopillar. With the increase of the diameter of the nanopillar, optical bandgap decreases and absorptance increases.

The influence of Xe ion irradiation on the optical bandgap was also investigated. The bandgap decreases with the increase of irradiation dose. It may be induced by the recrystallization during the irradiation and lead to the change in grain size, which is closely related to the bandgap of the film.

## Abbreviations

GLAD: glancing angle deposition; PASiNP: periodically aligned silicon nanopillar; PS: polystyrene; SEM: scanning electron microscopy; XRD: X-ray diffraction.

## Competing interests

The authors declare that they have no competing interests.

## Authors’ contributions

FY carried out the studies and drafted the manuscript. ZL participated in the design of the study and helped revise the manuscript. TZ participated in the experiments and data analysis. WM and ZZ gave suggestions on the analysis of results. All the authors read and approved the final manuscript.

## Authors’ information

All authors belong to the School of Materials Science and Engineering, Tsinghua University, People's Republic of China. FY is a master candidate interested in amorphous silicon thin film. ZL is an associate professor whose research fields include thin film material and nuclear material. TZ is a master candidate interested in the fabrication of nanostructure. WM is an associate professor working on nanostructure characterization. ZZ is the school dean professor with research interest in nanostructures and SERS effect.
